# High‐Concentration Hydrogen Peroxide Gels Containing Calcium Polyphosphate Associated With Violet Led Light: Bleaching Efficacy and Effects on Enamel Properties

**DOI:** 10.1111/jerd.70112

**Published:** 2026-01-18

**Authors:** Hemanuelly Albuquerque dos Anjos, Débora Alves Nunes Leite Lima, Carolina Meneghin Barbosa, Klaus Rischka, Waldemir Francisco Vieira‐Junior

**Affiliations:** ^1^ Department of Restorative Dentistry, Faculdade de Odontologia de Piracicaba Universidade Estadual de Campinas São Paulo Brazil; ^2^ Fraunhofer Institute for Manufacturing Technology and Advanced Materials (IFAM) Bremen Germany

**Keywords:** calcium compounds, phototherapy, tooth bleaching

## Abstract

**Objectives:**

To evaluate the effect of violet LED light (VL) and hydrogen peroxide (HP) in‐office bleaching gels supplemented with calcium polyphosphate (CaPP) or calcium gluconate (GU):

**Materials and Methods:**

Bovine enamel blocks (*n* = 10) were randomly assigned: commercial HP gel, commercial HP gel with GU, or experimental 35% HP with or without CaPP (0.5 or 1.5 wt%), and their VL‐irradiated versions. Bleaching efficacy was assessed by color analyses (CIE L*a*b*, Δ*E*_*ab*_, Δ*E*_00_, Δ*WID*) at baseline (*T*_0_) and after treatment (*T*_1_). Knoop microhardness (KHN), percentage surface microhardness loss (%SHL), and roughness variation (ΔRa) were measured at *T*_0_, *T*_1_, and 14 days post‐treatment (*T*_2_). Surface morphology was assessed by scanning electron microscopy (SEM). Data were analyzed using generalized linear models, Kruskal–Wallis and Dunn tests (*α* = 0.05).

**Results:**

The group irradiated only with VL exhibited lower color change (*p* < 0.0001). CaPP addition significantly increased Δ*E*_00_ in VL‐irradiated groups (*p* < 0.05). For roughness and SEM, no significant differences were observed among groups. Only CaPP‐ and GU‐containing gels restored initial KHN values at *T*_2_ (*p* < 0.05). CaPP groups showed lower %SHL than control groups (*p* < 0.05).

**Conclusions:**

Bleaching gels containing CaPP maintained bleaching efficacy and mediated enamel microhardness recovery. VL further enhanced the bleaching effect in CaPP gels.

**Clinical Significance:**

CaPP represents a promising alternative for bleaching gel formulations, promoting enamel remineralization and enhancing bleaching efficacy when combined with VL.

## 1 Introduction

Tooth bleaching is a widely accepted esthetic procedure that offers significant improvements in tooth color in a minimally invasive manner, without the removal of enamel or dentin [1, 2]. In general, tooth discoloration compromises the harmony and attractiveness of the smile, explaining the growing demand for this intervention [3].

The bleaching mechanism is based on the decomposition of hydrogen peroxide (HP), which releases reactive oxygen species capable of removing chromogenic staining, resulting in the whitening effect [4, 5]. However, free radicals can interact with dental hard tissues and the pulp, potentially causing risks such as tooth sensitivity, decreased microhardness, increased surface roughness, and structural alterations in enamel, which are influenced by the composition and pH of bleaching products [6, 7, 8, 9, 10, 11, 12, 13, 14]. Given these effects, calcium supplementation strategies have been explored to enhance the safety of bleaching protocols [12, 15, 16, 17, 18, 19, 20, 21] due to their chemical properties [22, 23]. Among the available additives, calcium gluconate has been incorporated into commercial gels, although its clinical benefits remain controversial [10, 12, 14, 15, 17, 18, 19, 21].

Calcium polyphosphate (CaPP) particles have emerged as a promising bioactive approach [24, 25, 26, 27, 28]. Upon degradation, CaPP releases phosphate ions, which bind to calcium cations on the tooth surface, promoting supersaturation and facilitating hydroxyapatite formation, thereby favoring remineralization [24, 25, 26, 27, 28]. Additionally, its long‐chain polymeric structure offers greater pH stability and biological activity than other calcium phosphates [24, 25]. Previous studies [26, 27, 28] demonstrated that CaPP reduces microhardness loss and maintains the physicochemical properties of bleached enamel in both low‐ and high‐concentration HP formulations. However, no studies have investigated the interaction of CaPP in high‐concentration gels combined with violet LED light (VL) activation or compared its effects with commercial gels containing calcium gluconate.

VL (405–410 nm) is a commonly used auxiliary source in clinical practice and has been proposed as a bleaching enhancer due to its overlap with the absorption peak of chromophore molecules of the chromogenic staining, which is characterized by the deposition of exogenous pigments with high molecular conjugation onto the enamel surface [29, 30, 31]. Although some reports suggest a mild standalone whitening effect, evidence on its actual efficacy, particularly in combination with gel formulations, remains limited and controversial [32, 33, 34, 35, 36, 37]. In this context, light exposure may stimulate the formation of precipitates on the enamel surface [38] and affect the interaction of CaPP particles with dental tissues, as previously observed for other calcium phosphates exposed to different light sources [39]. This hypothesis underscores the need to investigate the potential synergistic or modifying effects of this combination.

Therefore, the aim of this study was to evaluate whether VL activation, applied in combination with gels containing calcium gluconate or CaPP, impacts bleaching efficacy and the physical properties of enamel. The research hypotheses tested were: (1) VL activation of high‐concentration bleaching gels, with or without CaPP, enhances bleaching efficacy; (2) VL and bleaching gels containing CaPP influence enamel surface roughness after bleaching; and (3) bleaching gels with CaPP promote enamel surface microhardness that is more susceptible to recovery.

## 2 Materials and Methods

### 2.1 Study Design

**FIGURE 1:**
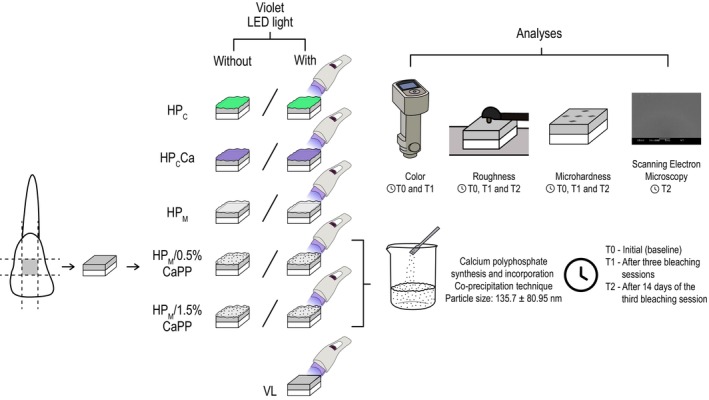
Experimental design. See Table 1 for abbreviations of groups. C, commercial product; Ca, product with calcium gluconate; CaPP, product with calcium polyphosphate; HP, 35% hydrogen peroxide; M, manipulated product; VL, violet LED light.

The experimental design of this in vitro study is illustrated in Figure 1. Bovine enamel specimens (4 × 4 × 2 mm) were randomly allocated to 11 treatment groups (*n* = 10) as follows: a conventional commercial in‐office bleaching gel (HP Maxx, FGM), a commercial gel containing calcium gluconate (HP Blue, FGM), and experimental gels containing 35% HP manipulated with or without calcium polyphosphate (CaPP) at concentrations of 0.5% or 1.5%. Each bleaching gel formulation was tested with and without VL irradiation. Additionally, a non‐bleached control group irradiated with VL was included.

Sample size was calculated using G*Power software (version 3.1.5, Heine, Universität Düsseldorf, Germany), considering 11 groups, *α* = 0.05, power (1 − *β*) = 0.90, and an effect size of 0.50 [40]. The analysis indicated a minimum sample size of *n* = 9. Therefore, the study was conducted with *n* = 10 per group, consistent with previous studies [27, 41].

Bleaching was performed in three sessions of 30 min each, separated by 7‐day intervals. VL irradiation (Bright Max Whitening, MMOptics) was applied in 20 cycles of 1 min each, with 30‐s intervals between cycles. Bleaching effectiveness was assessed by color analysis (CIE Lab*, Δ*E*_*ab*_, Δ*E*_00_, and ΔWID) at baseline (*T*_0_) and 24 h after the third bleaching session (*T*_1_) [27].

Knoop microhardness (KHN), percentage surface microhardness loss (%SHL), and surface roughness variation (ΔRa) were evaluated at *T*_0_, *T*_1_, and 14 days after the final bleaching session (*T*_2_) [42]. At *T*_2_, representative enamel specimens were analyzed by scanning electron microscopy (SEM) to characterize surface morphology.

### 2.2 Synthesis of CaPP

Amorphous CaPP sub‐microparticles were obtained using the co‐precipitation technique [43]. Sodium polyphosphate, with an average chain length of 40 phosphate units, was supplied by Chemische Fabrik Budenheim (Budenheim, Germany). One gram of sodium polyphosphate was dissolved in 50 mL of distilled water, and the pH was adjusted to 10 using 1 M sodium hydroxide solution.

A Ca/P ratio of 2:1 was used to obtain smaller particle sizes. Calcium chloride dihydrate (2.8 g, Sigma‐Aldrich, Taufkirchen, Germany) diluted in 50 mL of 96% ethanol was added dropwise (1 mL/min) to sodium polyphosphate solution, and pH was adjusted to 10 using 1 M NaOH at room temperature. The suspension was stirred for 4 h. The resulting CaPP particles were collected and washed three times with absolute ethanol. They were then dried at 60°C and sieved through an AS 200 mesh (100 μm; Retsch GmbH, Haan, Germany) [24, 26]. The average particle size was 135.7 ± 80.95 nm [26].

### 2.3 Bleaching Gels: Acquisition and Manipulation

Control gels: The commercial gels used included Whiteness HP Maxx (35% HP, FGM, Joinville, SC, Brazil) and Whiteness HP Blue (35% hydrogen peroxide with calcium gluconate, FGM, SC, Brazil). Additionally, a manipulated gel containing 35% HP, with the same base composition as the experimental gels but without CaPP, was tested. All gels were refrigerated (8°C ± 1°C) throughout the experiment.

Experimental gels: The base formulation consisted of 35% HP, glycerin, distilled water, carbopol 940, sodium hydroxide, and propylene glycol. Gels were stirred in the dark for 2 h at 9°C, while covered with aluminum foil to minimize peroxide degradation, until homogeneous. In the CaPP groups, two concentrations (0.5 and 1.5 wt%) were incorporated to evaluate the effect of CaPP concentration, as they do not significantly alter the viscosity or pH of the manipulated gel [26]. A NaOH‐based neutralizing solution was used to adjust the pH to neutrality.

### 2.4 Sample Preparation

Bovine teeth were collected in accordance with national and institutional ethical regulations. Teeth were examined under 10× magnification (Carl Zeiss, Santo Amaro, SP, Brazil) to detect caries, cracks, or enamel defects. Damaged teeth were discarded, and intact teeth were cleaned and stored in 0.1% thymol solution at 4°C [7].

Roots were sectioned 2 mm apical to the cementoenamel junction using a double‐faced diamond disc (KG Sorensen Ind. Com, Cotia, SP, Brazil) attached to a low‐speed handpiece under water irrigation. Enamel blocks (4 × 4 × 2.2 mm) were sectioned from the crowns using a diamond blade mounted on a precision cutter (Isomet 1000, Buehler, Lake Bluff, IL, USA).

Specimens were flattened and polished using sequential silicon carbide papers (p600, p1200, p2500, and p4000 grit; Buehler, Lake Bluff, IL, USA) on a polishing machine (Arotec SA, Cotia, SP, Brazil) under low speed and water cooling. Final polishing was performed with felt discs and diamond pastes (1 and 1/4 μm) under water cooling. Between steps, ultrasonic cleaning (Marconi, Piracicaba, SP, Brazil) was performed in distilled water for 10 min to remove debris and the smear layer.

Dentin was isolated using a clear acid‐resistant varnish (L'Apogée Alfaparf, Campo Grande, RJ, Brazil), leaving only the enamel surface exposed. Samples were then stained by immersion in black tea solution (Dr. Oetker, São Paulo, SP, Brazil) for 6 days at 37°C [28], with daily replacement of the solution. After staining, specimens were rinsed and stored in remineralizing solution for 1 week to stabilize the color.

### 2.5 Randomization and Bleaching Treatment

Samples were randomized into 11 treatment groups for each analysis (*n* = 10) using randomization tool (https://www.randomizer.org/). Experimental groups and gel/VL application protocols are shown in Table 1. Dental bleaching was carried out in 30‐min sessions [36], and repeated over three sessions with 7‐day intervals [7, 12].

**TABLE 1:** Experimental groups, gel compositions, and description of application method.

Group ID	Group	Application method	
		Gel/composition	VL
HP_C_	Commercial 35% HP (Whiteness HP MAXX)	30 min, 3 sessions 35% HP, thickeners, colorant blend, glycol, inorganic filler, deionized water^a^	Absent
HP_C_/VL	Commercial 35% HP (Whiteness HP MAXX) + violet LED light		20 cycles of 1 min each, with 30‐s intervals between cycles
HP_C_Ca	Commercial 35% HP with calcium gluconate (Whiteness HP Blue)	30 min, 3 sessions 35% HP, thickeners, inert violet pigment, neutralizing agents, calcium gluconate, glycol, deionized water^a^	Absent
HP_C_Ca/VL	Commercial 35% HP with calcium gluconate (Whiteness HP Blue) + violet LED light		20 cycles of 1 min each, with 30‐s intervals between cycles
HP_M_	35% HP (manipulated)	30 min, 3 sessions 35% HP, glycerin, distilled water, Carbopol 940, NaOH, propylene glycol	Absent
HP_M_/VL	35% HP (manipulated) + violet LED light		20 cycles of 1 min each, with 30‐s intervals between cycles
HP_M_/0.5% CaPP	35% HP with 0.5 wt% CaPP	30 min, 3 sessions 35% HP, 0.5 wt% CaPP, glycerine, distilled water, Carbopol 940, NaOH, propylene glycol	Absent
HP_M_/0.5% CaPP/VL	35% HP with 0.5 wt% CaPP + violet LED light		20 cycles of 1 min each, with 30‐s intervals between cycles
HP_M_/1.5% CaPP	35% HP with 1.5 wt% CaPP	30 min, 3 sessions 35% HP, 1.5 wt% CaPP, glycerin, distilled water, Carbopol 940, NaOH, propylene glycol	Absent
HP_M_/1.5% CaPP/VL	35% HP with 1.5 wt% CaPP + violet LED light		20 cycles of 1 min each, with 30‐s intervals between cycles
VL	Violet LED light	Absent	20 cycles of 1 min each, with 30‐s intervals between cycles

^a^ As provided by the manufacturer.

Specimens were kept in remineralizing solution at 37°C, replaced daily, starting 24 h before the first session and continuing between sessions. This solution simulates the inorganic effects of human saliva and contains 1.5 mM Ca, 0.9 mM P, 150 mM KCl, and 0.1 M Tris buffer at pH 7.0 [44].

The VL used was from a Bright Max Whitening device (MMOptics, São Carlos, SP, Brazil), delivering a total power of 1.2 W via four 300‐mW violet LEDs. The emitted wavelength ranged from 405 to 410 nm, with irradiance of 112 mW/cm^2^, application area of 10.7 cm^2^, and total session energy of 1440 J [45]. Irradiation was performed in 20 cycles of 1 min each, with 30‐s intervals between cycles, maintaining an 8 mm distance from the tooth surface, as per the manufacturer's instructions.

### 2.6 pH Analysis

Both commercial and experimental bleaching gels were analyzed for pH using a digital benchtop pH‐meter (PHS‐3B, Guangdong, China) calibrated with standard buffer solutions (pH 4.0 and 7.0) prior to testing. Each gel was evaluated in triplicate at 0, 5, 15, and 30 min. The electrode remained immersed in the gel throughout the measurement to prevent bubble formation.

### 2.7 Bleaching Efficacy: Color Measurement

Color was measured using a spectrophotometer (CM 700d, Minolta, Osaka, Japan). Spectral distribution was evaluated based on the CIE L*a*b* color system using On Color software (Konica Minolta, Osaka, Japan), where L* represents the lightness axis (white–black), a* the green–red axis, and b* the blue–yellow axis. The spectrophotometer had been previously calibrated using white and black reflectance standards, following the manufacturer's instructions. Measurements were taken at baseline and 24 h after the third bleaching session [7, 12, 41].

Differences in *L*,*a*, and b* values between timepoints were used to express overall color changes (Δ*E*_*ab*_ and Δ*E*_00_) [46]. Additionally, the variation in the Whiteness Index for Dentistry (WID) was used to quantify bleaching effectiveness [47]. The following formulas were applied:

$$I.\Delta E_{\mathrm{ab}}=\left[{\left(L_{1}-L_{0}\right)}^{2}+{\left(a_{1}-a_{0}\right)}^{2}+{\left(b_{1}-b_{0}\right)}^{2}\right]½$$

$$\begin{array}{c} \mathrm{II}.\Delta E_{00}\\ =\sqrt{{\left(\frac{\mathrm{\Delta L}}{K_{2}S_{2}}\right)}^{2}+{\left(\frac{\mathrm{\Delta C}}{K_{C}S_{C}}\right)}^{2}+{\left(\frac{\mathrm{\Delta H}}{K_{H}S_{H}}\right)}^{2}+R_{r}{\left(\frac{\mathrm{\Delta C}}{K_{C}S_{C}}\right)}^{2}{\left(\frac{\mathrm{\Delta H}}{K_{H}S_{H}}\right)}^{2}} \end{array}$$

$$\mathrm{III}.\mathrm{WID}=0.511L^{*}-2.324a^{*}-1.100b^{*}$$

a.ΔWID=WID₁–WID₀.

### 2.8 Surface Roughness

To assess the remineralization potential, surface roughness and microhardness were analyzed at three timepoints: baseline, 24 h after the final bleaching session, and 14 days after bleaching [42]. This approach allowed for evaluating the impact of gel composition on enamel physical properties during bleaching, as well as the potential of each gel to enhance mineral recovery post‐treatment [42].

A surface roughness tester (Mitutoyo SJ‐410 V.1.014, São Paulo, SP, Brazil) performed three scans over 3.0 mm of each specimen in different positions, with the stylus passing through the geometric center. Position changes were achieved by rotating the specimen 120°. The Ra value represents the average distance between peaks and valleys (in μm). The mean of the three readings was recorded as the final surface roughness of each specimen. The Ra values were evaluated considering acceptable clinical limits [48].

### 2.9 Knoop Microhardness

Surface microhardness (KHN, kgf/mm^2^) was evaluated using a microhardness tester (HMV‐2 T E, Shimadzu, Tokyo, Japan) with a Knoop indenter, applying a 50 g load for 5 s [9]. Measurements were taken at baseline, 24 h after the final bleaching session, and 14 days after bleaching. At each time point, five indentations were performed per sample: one in the center and four at 100 μm from the center. The average of the five values was recorded as the final KHN value for each specimen [9]. Surface hardness loss percentage (%SHL) was calculated using the following formula:

$$I.\%\mathrm{SHL}=\left[\left({\mathrm{KHN}}_{i}-{\mathrm{KHN}}_{f}\right)/{\mathrm{KHN}}_{i}\right]\times 100$$

where KHN_i_ is the initial mean microhardness, and KHN_f_ is the final mean microhardness.

### 2.10 Scanning Electron Microscopy (SEM)

At the end of the study, three randomly selected specimens from each group were qualitatively analyzed by SEM (JEOL JSM 5600LV, Tokyo, Japan). Untreated enamel specimens were used as a negative control. Samples were ultrasonically cleaned (Marconi, Piracicaba, SP, Brazil), then sequentially dehydrated in absolute alcohol solutions (50%, 75%, 95%, and 100%) for 20 min each. They were then mounted on acrylic stubs and sputter‐coated with a thin layer of gold–palladium (Bal‐Tec SCD 050 sputter coater, Germany). Representative areas of each group were imaged at 4000× magnification.

### 2.11 Statistical Analysis

Descriptive and exploratory analyses were initially performed. Generalized linear mixed models for repeated measures were used to analyze the effects of treatment group, time, and their interaction on L* and a* coordinates. Generalized linear models were used to assess the effect of treatment group on overall color change (Δ*E*_*ab*_ and Δ*E*_00_). The b* coordinate and microhardness (KHN) data were analyzed using the Kruskal‐Wallis and Dunn tests for group comparisons, and the Friedman and Nemenyi tests for time comparisons. Surface roughness variation (ΔRa), microhardness loss (%SHL), and ΔWID were analyzed using the Kruskal‐Wallis and Dunn tests. All statistical analyses were performed using R software (R Core Team, 2025), with a significance level set at 5%.

## 3 Results

The initial pH values of the bleaching gels used in this study ranged from 6.18 to 6.62 (Table 2). Overall, the gels remained close to neutrality at all time points, and a gradual increase in pH was observed over time. The commercial gels (HP_C_ and HP_C_Ca) showed slightly higher pH values than the manipulated experimental gels. The bleaching gels containing CaPP (0.5% or 1.5%) presented similar pH values to the manipulated bleaching gel without CaPP (HP_M_) at all evaluation times.

**TABLE 2:** Mean (standard deviation) pH values for bleaching gels.

Bleaching gel	Time (min)			
	0	5	15	30
HP_C_	6.62 (0.02)	6.82 (0.04)	6.93 (0.04)	7.09 (0.02)
HP_C_Ca	6.44 (0.05)	6.68 (0.03)	6.69 (0.06)	6.87 (0.03)
HP_M_	6.17 (0.03)	6.39 (0.02)	6.52 (0.05)	6.68 (0.05)
HP_M_/0.5% CaPP	6.25 (0.05)	6.32 (0.01)	6.47 (0.03)	6.60 (0.04)
HP_M_/1.5% CaPP	6.18 (0.05)	6.29 (0.03)	6.52 (0.04)	6.52 (0.03)

*Note:* See Table 1 for group IDs: HP = 35% hydrogen peroxide, C = commercial product, M = manipulated product, Ca = product with calcium gluconate, CaPP = product with calcium polyphosphate.

The results for the L* coordinate are presented in Table 3. Statistical analysis indicated *p* = 0.3856 for the treatment group factor, *p* < 0.0001 for the time factor, and *p* = 0.0556 for the interaction. Accordingly, no significant differences were found between groups for baseline L* values (*p* > 0.05). At the end of treatment, all groups showed an increase in L* values compared with baseline (*p <* 0.05), except for the VL group (*p* > 0.05). The VL group, which was exposed only to violet LED light, exhibited the lowest L* values and differed significantly from the other treatment groups (*p* < 0.05). Groups HP_M_/VL and HP_M_/0.5% CaPP/VL showed higher L* values and were statistically different and superior to the HP_M_/1.5% CaPP group (*p* < 0.05).

**TABLE 3:** Mean (standard deviation) of the L* coordinate for different bleaching treatments and times.

	Time	
	Initial	After 3rd session
HP_C_	69.40 (2.42)^Ba^	76.41 (2.47)^Aab^
HP_C_/VL	69.50 (2.44)^Ba^	77.48 (3.80)^Aab^
HP_C_Ca	69.55 (2.59)^Ba^	76.56 (2.11)^Aab^
HP_C_Ca/VL	69.56 (2.62)^Ba^	77.14 (2.26)^Aab^
HP_M_	69.59 (2.71)^Ba^	76.75 (1.87)^Aab^
HP_M_/VL	69.71 (2.68)^Ba^	77.59 (1.90)^Aa^
HP_M_/0.5% CaPP	69.71 (2.64)^Ba^	75.86 (3.24)^Aab^
HP_M_/0.5% CaPP/VL	69.70 (2.62)^Ba^	77.68 (1.88)^Aa^
HP_M_/1.5% CaPP	69.68 (2.51)^Ba^	75.74 (2.08)^Ab^
HP_M_/1.5% CaPP/VL	69.51 (2.02)^Ba^	77.79 (2.93)^Aab^
VL	69.55 (2.02)^Aa^	70.50 (2.14)^Ac^

*Note:* Different letters (uppercase in rows and lowercase in columns) indicate statistically significant differences (*p ≤* 0.05). Mixed‐effects generalized linear models for repeated measures: *p*(group) = 0.3856; *p*(time) < 0.0001; *p*(interaction) = 0.0556.

Abbreviations: C, commercial product; Ca, product containing calcium gluconate; CaPP, product with calcium polyphosphate; HP, 35% hydrogen peroxide; M, compounded product; VL, violet LED light.

The results for the a* coordinate are presented in Table 4, with *p*‐values of 0.1409 for the treatment group factor, < 0.0001 for the time factor, and 0.0005 for the interaction. No statistically significant differences were observed among the groups for baseline a* values (*p* > 0.05). After the third bleaching session, all groups showed a reduction in a* values compared with baseline (*p* < 0.05), except for the VL group, which showed an increase in a* values (*p* < 0.05) and differed significantly from the other treatment groups (*p* < 0.05). HP_C_Ca and HP_M_ presented lower a* values than their respective irradiated versions, HP_C_Ca/VL and HP_M_/VL.

**TABLE 4:** Mean (standard deviation) of the a* coordinate for different bleaching treatments and times.

	Time	
	Initial	After 3rd session
HP_C_	2.88 (0.85)^Aa^	1.42 (0.46)^Bde^
HP_C_/VL	3.02 (0.78)^Aa^	1.68 (0.49)^Bbcde^
HP_C_Ca	2.51 (0.85)^Aa^	1.57 (0.41)^Bcde^
HP_C_Ca/VL	2.61 (0.70)^Aa^	1.97 (0.21)^Bb^
HP_M_	2.51 (0.82)^Aa^	1.33 (0.37)^Be^
HP_M_/VL	2.73 (0.62)^Aa^	1.84 (0.34)^Bbc^
HP_M_/0.5% CaPP	2.30 (0.75)^Aa^	1.49 (0.69)^Bcde^
HP_M_/0.5% CaPP/VL	2.55 (0.70)^Aa^	1.71 (0.45)^Bbcd^
HP_M_/1.5% CaPP	2.91 (1.18)^Aa^	1.63 (0.48)^Bbcde^
HP_M_/1.5% CaPP/VL	2.63 (0.62)^Aa^	1.59 (0.53)^Bbcde^
VL	2.38 (0.81)^Ba^	2.80 (0.56)^Aa^

*Note:* Different letters (uppercase in rows and lowercase in columns) indicate statistically significant differences (*p* ≤ 0.05). Mixed‐effects generalized linear models for repeated measures: *p*(group) = 0.1409; *p*(time) < 0.0001; *p*(interaction) = 0.0005.

Abbreviations: C, commercial product; Ca, product containing calcium gluconate; CaPP, product with calcium polyphosphate; HP, 35% hydrogen peroxide; M, compounded product; VL, violet LED light.

The results for the b* coordinate are presented in Table 5. No statistically significant differences were observed among the groups at baseline (*p* = 0.1960). After the third bleaching session, all groups showed a reduction in b* values compared with baseline (*p* < 0.05), except for the VL group, which maintained similar values (*p* > 0.05). Furthermore, the VL group exhibited significantly higher b* values (*p* < 0.0001) than all other groups, except for HP_C_, HP_C_Ca, and HP_M_/1.5% CaPP (*p* > 0.05).

**TABLE 5:** Median (minimum; maximum) of the b* coordinate for different bleaching treatments and times.

Group	Time		*p*
	Initial	After 3rd session	
HP_C_	9.43 (7.78; 14.97)^Aa^	3.89 (1.65; 7.04)^Bab^	0.0051
HP_C_/VL	11.05 (6.26; 14.68)^Aa^	2.96 (1.01; 4.37)^Bb^	0.0051
HP_C_Ca	8.38 (6.39; 11.87)^Aa^	4.69 (3.32; 7.68)^Bab^	0.0051
HP_C_Ca/VL	9.15 (6.13; 11.10)^Aa^	3.99 (1.32; 5.79)^Bb^	0.0051
HP_M_	9.52 (4.37; 12.59)^Aa^	3.39 (1.83; 6.45)^Bb^	0.0051
HP_M_/VL	8.78 (5.65; 12.77)^Aa^	2.82 (0.64; 5.66)^Bb^	0.0051
HP_M_/0.5% CaPP	7.62 (6.46; 11.82)^Aa^	3.53 (1.55; 5.72)^Bb^	0.0051
HP_M_/0.5% CaPP/VL	8.54 (6.36; 12.45)^Aa^	2.03 (−1.06; 5.63)^Bb^	0.0051
HP_M_/1.5% CaPP	9.49 (6.19; 13.15)^Aa^	5.04 (1.11; 10.18)^Bab^	0.0051
HP_M_/1.5% CaPP/VL	9.87 (7.77; 15.46)^Aa^	2.15 (1.33; 5.43)^Bb^	0.0051
VL	8.03 (5.40; 13.49)^Aa^	8.48 (5.66; 14.12)^Aa^	0.2411
*p*	0.1960	< 0.0001	

*Note:* Different letters (uppercase in rows and lowercase in columns) indicate statistically significant differences (*p* ≤ 0.05).

Abbreviations: C, commercial product; Ca, product containing calcium gluconate; CaPP, product with calcium polyphosphate; HP, 35% hydrogen peroxide; M, compounded product; VL, violet LED light.

According to the results for overall color change (Table 6), both Δ*E*_*ab*_ and Δ*E*_00_ values were significantly lower in samples from the group irradiated with violet LED light only (VL) than for all other bleaching groups (*p* < 0.0001). Comparing the bleaching gels and their irradiated counterparts, only the HP_M_/1.5% CaPP/VL group showed significantly higher Δ*E*_*ab*_ values than its non‐irradiated version (HP_M_/1.5% CaPP; *p* < 0.05). However, for Δ*E*_00_, both CaPP concentrations (0.5% and 1.5%) showed significantly higher values in the irradiated groups (*p* < 0.05). Additionally, bleaching gel groups without VL did not show statistically significant differences in either Δ*E*_*ab*_ or Δ*E*_00_ among themselves (*p* > 0.05). The Δ*WID* results (Table 6) showed statistical differences only between the groups treated with bleaching gel, which showed higher and positive values, and the group irradiated with violet LED only (*p* < 0.0001), except for HP_C_Ca, HP_M_/0.5% CaPP, and HP_M_/1.5% CaPP, which did not differ from any of the other experimental groups.

**TABLE 6:** Mean (standard deviation) values for overall color change (Δ*E*_ab_ and Δ*E*_00_) and median (minimum, maximum) values for ΔWID.

Group	Variable		
	Δ*E*_ab_	Δ*E*_00_	ΔWID
HP_C_	9.34 (2.11)^ab^	7.06 (1.46)^abc^	12.1 (8.8, 18.8)^a^
HP_C_/VL	11.75 (3.53)^a^	8.83 (2.52)^a^	15.7 (8.7, 22.6)^a^
HP_C_Ca	8.18 (2.46)^b^	6.20 (1.88)^bc^	9.5 (5.4, 18.6)^ab^
HP_C_Ca/VL	9.50 (2.48)^ab^	7.23 (1.82)^abc^	11.0 (5.3, 18.6)^a^
HP_M_	9.09 (2.91)^ab^	6.89 (2.13)^abc^	11.3 (5.4, 18.5)^a^
HP_M_/VL	10.33 (1.40)^ab^	7.91 (1.05)^abc^	12.1 (10.6, 17.9)^a^
HP_M_/0.5% CaPP	7.94 (3.15)^b^	6.13 (2.26)^c^	10.0 (0.7, 15.9)^ab^
HP_M_/0.5% CaPP/VL	10.57 (1.35)^ab^	8.16 (0.89)^ab^	13.2 (11.2, 16.6)^a^
HP_M_/1.5% CaPP	7.90 (1.72)^b^	6.07 (1.26)^c^	11.3 (8.3, 17.6)^ab^
HP_M_/1.5% CaPP/VL	11.40 (3.77)^a^	8.63 (2.71)^a^	14.8 (4.9, 24.8)^a^
VL	2.18 (1.50)^c^	1.77 (1.09)^d^	−0.8 (−4.4, 1.1)^b^
*p*	< 0.0001	< 0.0001	< 0.0001

*Note:* Different letters in columns indicate statistically significant differences (*p* ≤ 0.05).

Abbreviations: C, commercial product; Ca, product containing calcium gluconate; CaPP, product with calcium polyphosphate; HP, 35% hydrogen peroxide; M, compounded product; VL, violet LED light.

The results for surface roughness variation are shown in Table 7. The baseline Ra measurement indicated a mean of 0.027 μm with a standard deviation of 0.003. Overall, the change in roughness was close to zero across all groups, with no statistically significant differences detected when comparing baseline with the third bleaching session (*p* = 0.145) or with 14 days post‐treatment (*p* = 0.184).

**TABLE 7:** Median (minimum; maximum) of surface roughness variation (ΔRa) for different bleaching treatments and times.

Group	Comparison	
	Initial *x* after 3rd session	Initial *x* after 14 days
HP_C_	0.01 (−0.01; 0.02)^a^	0.01 (−0.01; 0.02)^a^
HP_C_/VL	0 (−0.01; 0.02)^a^	0 (−0.01; 0.02)^a^
HP_C_Ca	0 (−0.01; 0.01)^a^	−0.01 (−0.02; 0.01)^a^
HP_C_Ca/VL	0 (0; 0.01)^a^	0 (−0.01; 0)^a^
HP_M_	0 (−0.01; 0.01)^a^	0 (−0.01; 0.01)^a^
HP_M_/VL	0 (−0.01; 0.02)^a^	0 (−0.01; 0.03)^a^
HP_M_/0.5% CaPP	0 (−0.01; 0.02)^a^	0 (−0.01; 0.02)^a^
HP_M_/0.5% CaPP/VL	−0.01 (−0.01; 0.02)^a^	0 (−0.01; 0.01)^a^
HP_M_/1.5% CaPP	0 (−0.01; 0.02)^a^	0 (−0.01; 0.02)^a^
HP_M_/1.5% CaPP/VL	−0.01 (−0.01; 0.02)^a^	0 (−0.01; 0.02)^a^
VL	0 (−0.01; 0.04)^a^	0 (−0.01; 0.04)^a^
*p*	0.145	0.184

*Note:* Different letters in columns indicate statistically significant differences (*p* ≤ 0.05).

Abbreviations: C, commercial product; Ca, product containing calcium gluconate; CaPP, product with calcium polyphosphate; HP, 35% hydrogen peroxide; M, compounded product; VL, violet LED light.

The microhardness (KHN) results are presented in Table 8. No statistically significant differences were observed among the groups at baseline (*p* > 0.05). Comparing different timepoints, the bleached groups showed a decrease in microhardness after the third session (*p* < 0.0001), except for HP_C_, HP_C_/VL, and VL. However, HP_C_ and HP_C_/VL showed significantly lower values 14 days post‐treatment compared with baseline (*p* < 0.001), as did HP_M_. All groups supplemented with calcium, either calcium gluconate (Ca) or calcium polyphosphate (CaPP), showed no statistically significant differences between the initial and final values (*p* > 0.05).

**TABLE 8:** Median (minimum; maximum) of surface microhardness (KHN) for different bleaching treatments and times.

Group	Time			*p*
	Initial	After 3rd session	After 14 days	
HP_C_	352 (311; 385)^Aa^	299 (280; 327)^ABab^	267 (254; 298)^Bb^	< 0.0001
HP_C_/VL	355 (313; 386)^Aa^	310 (284; 325)^ABab^	273 (249; 301)^Bb^	< 0.0001
HP_C_Ca	359 (315; 387)^Aa^	248 (237; 268)^Bbcd^	325 (302; 359)^Ab^	0.0001
HP_C_Ca/VL	362 (319; 388)^Aa^	242 (224; 266)^Bcd^	337 (305; 356)^Aa^	0.0001
HP_M_	350 (312; 386)^Aa^	239 (228; 257)^Bd^	269 (252; 299)^Ba^	0.0001
HP_M_/VL	354 (315; 388)^Aa^	236 (223; 261)^Bd^	273 (254; 301)^ABb^	< 0.0001
HP_M_/0.5% CaPP	351 (311; 384)^Aa^	305 (294; 322)^Babc^	330 (307; 352)^Aab^	0.0012
HP_M_/0.5% CaPP/VL	341 (312; 387)^Aa^	313 (295; 327)^Ba^	336 (315; 353)^Aa^	0.0022
HP_M_/1.5% CaPP	331 (314; 365)^Aa^	314 (296; 328)^Ba^	339 (325; 355)^Aa^	0.0006
HP_M_/1.5% CaPP/VL	336 (318; 369)^Aa^	316 (298; 329)^Ba^	338 (320; 387)^Aa^	0.0006
VL	340 (321; 372)^Aa^	359 (315; 388)^Aa^	324 (313; 387)^Aa^	0.0821
*p*	0.4194	< 0.0001	< 0.0001	

*Note:* Different letters (uppercase for horizontal comparisons and lowercase for vertical comparisons) indicate statistically significant differences (*p* ≤ 0.05).

Abbreviations: C, commercial product; Ca, product containing calcium gluconate; CaPP, product with calcium polyphosphate; HP, 35% hydrogen peroxide; M, compounded product; VL, violet LED light.

In the between‐group comparison after the third session, all groups containing CaPP (0.5% or 1.5%) exhibited higher and statistically different microhardness values compared with HP_M_ (*p* < 0.05). After 14 days, three groups had the highest microhardness values among the bleaching gel treatments: HP_M_/0.5% CaPP/VL, HP_M_/1.5% CaPP, and HP_M_/1.5% CaPP/VL. These differed significantly from HP_C_, HP_C_Ca, and HP_M_ (*p* < 0.05). Finally, HP_C_Ca and HP_M_ showed lower microhardness values than their irradiated versions, HP_C_Ca/VL and HP_M_/VL, respectively.

**FIGURE 2:**
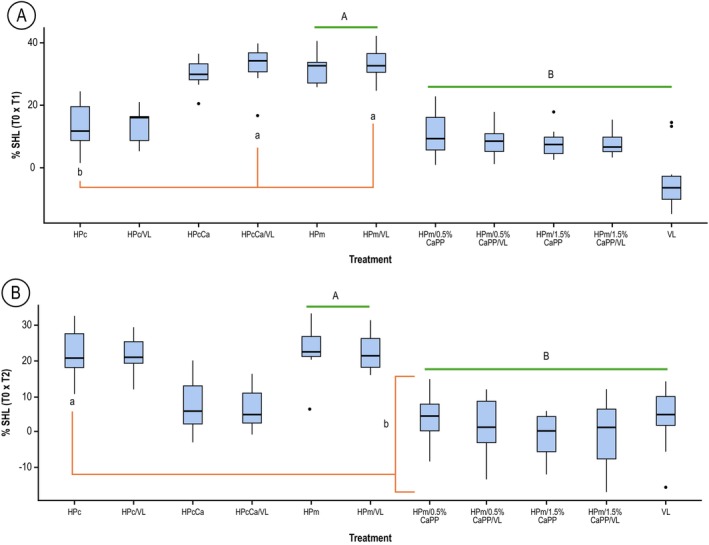
Boxplot of percentage surface microhardness loss (%SHL), comparing (A) baseline vs. after the third session and (B) baseline vs. 14 days post‐treatment. Legend: Uppercase letters indicate statistical differences compared with the HP_M_ and HP_M_/VL groups (compounded gel without remineralizing agents), and lowercase letters indicate statistical differences compared with HP_C_, representing the commercial bleaching gel without remineralizing agents. Kruskal–Wallis, *p* < 0.0001. Abbreviations as described in Table 1: HP = 35% hydrogen peroxide, VL = violet LED light, C = commercial product, M = compounded product, Ca = product containing calcium gluconate, CaPP = product containing calcium polyphosphate.

The results for percentage surface microhardness loss (%SHL, *p* < 0.0001) are shown in Figure 2. Comparing baseline with after the third bleaching session (boxplot A), it can be observed that HP_M_ and HP_M_/VL exhibited higher %SHL values than the groups containing CaPP, regardless of VL exposure. Additionally, HP_C_Ca/VL and HP_M_/VL showed significantly higher %SHL values than HP_C_ (*p* < 0.05). In the comparison after 14 days (boxplot B), groups containing CaPP (0.5% and 1.5%), with or without VL, as well as the VL‐only group, exhibited lower %SHL values than HP_C_, HP_M_, and HP_M_/VL (*p* < 0.05). No statistically significant differences were detected between the irradiated and non‐irradiated versions of the same bleaching gel.

**FIGURE 3:**
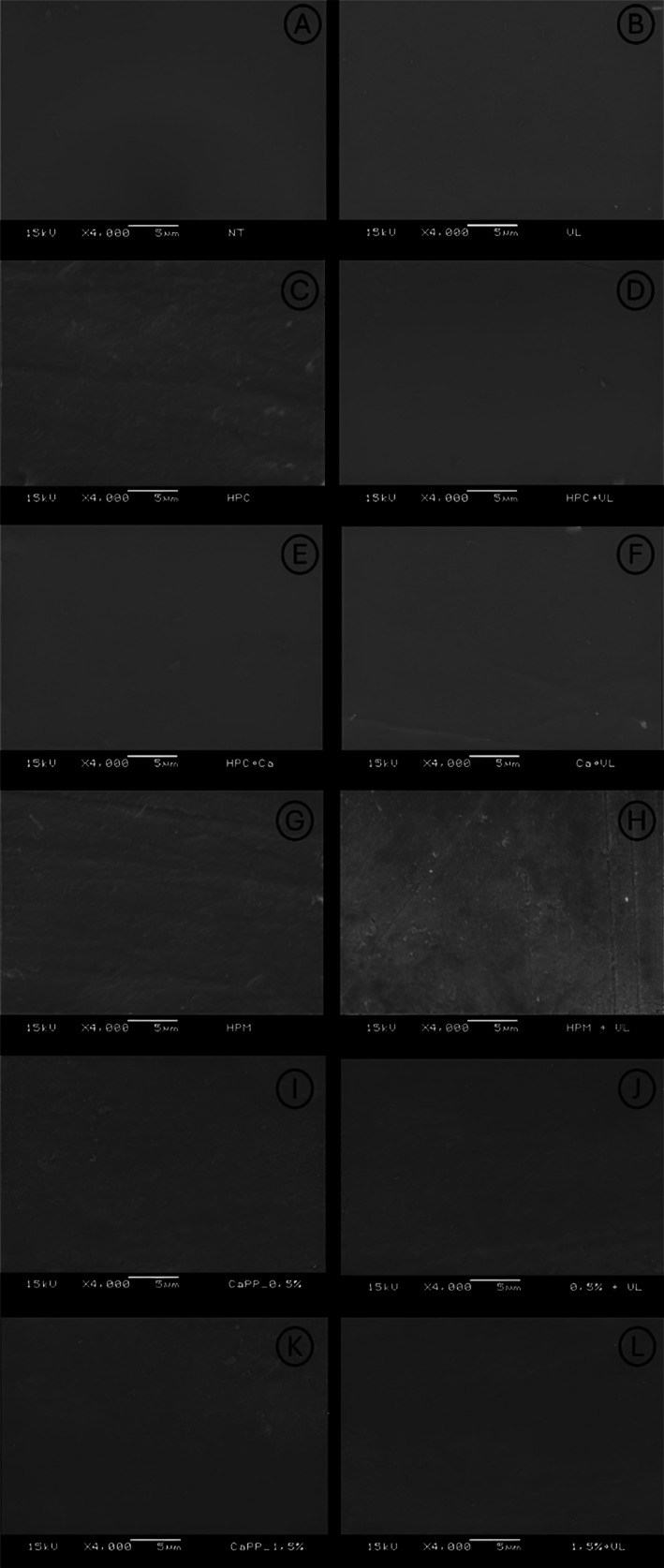
SEM images. (A) No treatment; (B) VL; (C) HP_C_; (D) HP_C_/VL; (E) HP_C_Ca; (F) HP_C_Ca/VL; (G) HP_M_; (H) HP_M_/VL; (I) HP_M_/0.5% CaPP; (J) HP_M_/0.5% CaPP/VL; (K) HP_M_/1.5% CaPP; (L) HP_M_/1.5% CaPP/VL. Abbreviations according to Table 1.

Figure 3 presents SEM images. Overall, all groups showed no or minimal morphological changes to the enamel surface. Samples treated with commercial gels (HP_C_ and HP_C_Ca) exhibited smooth and homogeneous surfaces, similar to the untreated enamel and the group irradiated with VL. In contrast, the samples treated with the compounded bleaching gel (HP_M_ and HP_M_/VL) showed depressions, pores, and signs of demineralization—alterations not observed in the groups containing CaPP (0.5% and 1.5%), regardless of VL exposure. Surface mineral precipitates, although not widespread, were detected in the groups treated with gels containing either calcium gluconate or CaPP.

## 4 Discussion

Although dental bleaching is considered an effective and safe treatment [2], the development of new formulations has been encouraged to reduce or control adverse effects [20, 21]. Therefore, the present study was designed to investigate the potential effects of CaPP in the formulation of new bleaching gels containing high‐concentration HP, in association with VL. The study was based on the premise of evaluating the bleaching efficacy and the impact of bleaching gels on enamel physical properties. The results partially accepted the hypothesis 1 because VL did not enhance the bleaching efficacy, but when combined with CaPP bleaching gels, it resulted in higher Δ*E*_00_ values than its non‐CaPP version.

The efficacy of all groups treated with bleaching gels, whether commercial or compounded, was demonstrated by increased L* values and decreased b* values, indicating lighter and less yellow samples post‐treatment [9, 12, 27, 42]. After three sessions, lower a* values were observed in the HP_C_, HP_C_Ca, and HP_M_ groups, indicating degradation of red pigments from black tea due to the oxidative action of hydrogen peroxide and reactive species [5]. Based on the thresholds for overall color change [46], all bleaching treatments resulted in clinically perceptible color changes (Δ*E*_*ab*_ > 1.2, Δ*E*_00_ > 0.8) and exceeded the acceptability thresholds (Δ*E*_*ab*_ > 2.7 and Δ*E*_00_ > 1.8). Moreover, Δ*WID* values surpassed the perceptibility and acceptability limits [47], demonstrating the increased brightness of the samples. In general, all bleached groups showed significant differences in color properties compared with the group exposed only to VL, which exhibited a limited bleaching effect.

VL (405–410 nm) can excite chromophore molecules in the dental structure, facilitating the breakdown of their chemical bonds and promoting bleaching [29, 30]. However, in the present study, VL alone did not achieve satisfactory whitening, as previously reported [30]. Factors such as shallow light penetration, number of sessions, and exposure protocol may explain these outcomes [30, 35, 36]. Furthermore, in gels without CaPP (HP_C_, HP_C_Ca, and HP_M_), VL did not enhance bleaching efficacy, as no differences were observed in color properties compared with their VL versions. VL appears to be more effective when combined with lower‐concentration bleaching gels [29, 33], where the release of free radicals is slower and less intense [34], than in high‐concentration gels with more intense and saturated radical release [37]. However, in gels with CaPP at both concentrations, Δ*E*_00_ values were higher with LED light exposure, and for Δ*E*_*ab*_, this was true at 1.5% CaPP.

It is hypothesized that VL combined with CaPP enhances bleaching performance by altering the decomposition pathway of HP, thereby increasing whitening effectiveness. Calcium saturation in the bleaching gel may influence the HP breakdown rate and the interfacial interaction between enamel and gel [15, 19]. The presence of calcium may stabilize the gel, slowing peroxide decomposition and radical release [19], as similarly reported for low‐concentration gels exposed to VL [34]. Additionally, VL acts as a photosensitive catalyst, and the gel's color or the CaPP itself may influence photoreactivity and radical formation. VL also slightly increases the gel's temperature [36], which could affect aggregation, recrystallization, and by‐product formation of CaPP [22]. However, these effects were not observed in the commercial gel containing calcium gluconate, possibly due to differences in gel pH, a factor known to influence peroxide decomposition and reactive oxygen species penetration into the tooth structure [13].

In contrast to the color changes observed, variations in surface roughness were negligible, leading to the rejection of hypothesis 2; similar behavior was observed across all experimental groups, regardless of light exposure or evaluation time. Previous studies also found no changes in enamel roughness after bleaching, even with calcium‐based additives in the gels [16, 27]. Generally, when the gel's pH is near neutral, bleaching‐induced roughness changes tend to be absent or minimal [12], remaining within clinically acceptable limits [48]. These findings are supported by the SEM images, which showed either no changes or minor alterations. Furthermore, initial topographic alterations may be reversed naturally by human saliva or remineralizing solutions in vitro [8, 27, 42].

However, enamel microhardness was affected by the bleaching protocol. Even though the gels had near‐neutral pH, specific radicals generated during peroxide decomposition (˙OH, ˙O_2_^−^, H^+^) can impact enamel properties [6]. High HP concentrations can promote calcium and phosphate ion loss, suggesting that radicals act on both the mineral and organic enamel matrix [4, 10, 11]. However, gels containing CaPP produced lower %SHL values and post‐treatment microhardness similar to that of untreated enamel, supporting hypothesis 3 concerning the promotion of mineral recovery by CaPP. Both CaPP concentrations (0.5% and 1.5%) resulted in better maintenance of microhardness over time, with no statistically significant differences from baseline values. These findings align with previous studies reporting CaPP ability to minimize microhardness loss during bleaching [27, 28]. The protective effects observed in CaPP‐treated groups may be due to its mechanisms of action. One mechanism involves direct binding, where phosphate groups in hydroxyapatite interact with CaPP calcium ions, forming strong precipitates on enamel [23, 43]. Another involves hydrolytic degradation of amorphous CaPP, releasing Ca^2+^ and PO_4_^3−^ ions, which precipitate and form an amorphous mineral layer on enamel [23, 24, 25, 26, 27]. This layer acts as a precursor for a more organized crystalline matrix resembling native hydroxyapatite [23, 25].

Thus, CaPP not only replenishes ions at the enamel surface but also promotes mineral recovery, explaining the higher microhardness values in CaPP‐supplemented groups after 14 days in remineralizing solution. The commercial gel with calcium gluconate also showed final Knoop values similar to the baseline, although no significant differences in %SHL were found for the HP_C_ group. The lower enamel dissolution and the presence of surface precipitates observed in SEM images in both CaPP and calcium gluconate groups may be due to the protective effects of calcium supplementation [21, 26, 28]. Calcium‐based agents contribute to the formation of an amorphous mineral layer that acts as a physical barrier against peroxide and promotes remineralization [26, 27]. These results align with previous findings that calcium supplementation during bleaching can reduce mineral loss and preserve enamel physicochemical properties [12, 26, 27].

The findings of this study indicate that the combination of violet light (VL) with CaPP‐containing bleaching gels represents a promising approach for dental bleaching. However, the study has limitations inherent to laboratory models, including the absence of human saliva as a storage medium, the use of a single LED exposure protocol, and the lack of evaluation of other calcium‐based agents. Moreover, color analyses were performed only after the last bleaching session, and the long‐term effects of CaPP‐containing bleaching gels should be addressed in future research. Future investigations should explore the bleaching efficacy of VL and CaPP combinations using lower hydrogen peroxide concentrations in in vivo and in situ models, and incorporate complementary methodologies for chemical and mechanical characterization. Additionally, further studies could examine the associative effects of CaPP with other remineralizing agents, as well as the impact of different concentrations.

## 5 Conclusion

Within the limitations of the present study, it was concluded that the incorporation of 0.5% and 1.5% CaPP into high‐concentration bleaching gels promoted enamel mineral recovery to levels comparable to unbleached enamel and, when combined with VL, enhanced the color change achieved by dental bleaching.

## Funding

This work was supported by Coordenação de Aperfeiçoamento de Pessoal de Nível Superior, Finance Code 001.

## Ethics Statement

This article does not contain any studies with human participants. Institutional Animal Care and Use Committee (no. 276/2025).

## Consent

The authors have nothing to report.

## Conflicts of Interest

The authors declare no conflicts of interest.

## Data Availability

The data that support the findings of this study are available from the corresponding author upon reasonable request.
